# Myelodysplastic syndrome-post cytotoxic therapy for pediatric low-grade glioma

**DOI:** 10.1007/s00381-025-06855-9

**Published:** 2025-05-29

**Authors:** Phoebe Power, Susannah Payne, Rebecca Walsh, Adam Nelson, Neevika Manoharan

**Affiliations:** 1 Kids Cancer Centre, Sydney Children’s Hospital, High Street, Randwick, NSW 2031 Australia; 2 Prince of Wales Hospital, Randwick, Australia; 3 New South Wales Health Pathology, Randwick, Australia; 4 School of Women’s and Children’s Health, UNSW Sydney, Kensington, Australia; 5 Children’s Cancer Institute, Kensington, Australia; 6 Present Address: Department of Pediatric Oncology, Dana-Farber/Boston Children’s Cancer and Blood Disorders Center, Boston, MA USA

**Keywords:** Pediatric low-grade glioma, Myeloid neoplasm-post cytotoxic therapy

## Abstract

Myeloid neoplasms-post cytotoxic therapy (MN-pCT, previously therapy-related myeloid neoplasms/tMN), are secondary malignancies associated with prior chemotherapy treatment, historically carrying a very poor prognosis. These are rarely associated with primary central nervous system (CNS) tumors, usually high-grade CNS malignancies requiring intensive multimodal treatment. Pediatric low-grade gliomas (pLGGs) are the most common childhood CNS tumors, and up to 50% of patients will require adjuvant therapy, which has traditionally consisted of low-dose metronomic chemotherapy, though the recent identification of key molecular drivers of pLGG means targeted therapies are changing this paradigm. We present a novel case of a 17-year-old girl with therapy-related myelodysplastic syndrome following chemotherapeutic treatment for pLGG. Given the poor prognosis of MN-pCTs, this case represents an important note of caution when choosing appropriate therapy for pLGG, especially considering the evolving role for targeted treatments in this disease.

## Introduction

Myeloid neoplasms-post cytotoxic therapy (MN-pCT, previously referred to as therapy-related myeloid neoplasms or tMNs), are rare but devastating secondary conditions arising in the setting of previous cytotoxic treatment for an unrelated malignancy [1–4]. They develop most commonly in the setting of previous alkylator or topoisomerase II inhibitor use and are therefore mostly associated with primary neoplasms that require intensive treatment with these agents, such as high-grade solid tumors of bone, soft tissue, and the CNS [3–6]. MN-pCT occur in 0.5–1% of children following cancer treatment and are associated with a worse prognosis than de novo pediatric myelodysplastic syndrome (MDS) or acute myeloid leukemia (AML), with 5-year overall survival (OS) of < 10% in untreated and ≤ 30% in treated patients [2, 3, 6–11].

Pediatric low grade gliomas (pLGGs) are the most common central nervous system (CNS) tumors of childhood and are associated with excellent long-term survival outcomes, (> 80% 10-year OS) [12, 13]. Whilst surgical resection can be curative, tumors occurring in less surgically accessible locations (such as the optic pathway or hypothalamus) often require medical treatment for disease control. This has traditionally consisted of low-dose metronomic-style chemotherapy, with combination vincristine-carboplatin or single agent vinblastine widely used as first-line therapy. These regimens achieve 5-year progression-free survival rates of around 45–55%, therefore many patients experience further progressions and require multiple lines of therapy [14, 15]. Recent advances in molecular profiling have led to a greater understanding of the molecular drivers of pLGG, most commonly alterations in the mitogen-activated protein kinase (MAPK) pathway [16–20]. This has led to the development and investigation of multiple novel targeted therapies which are changing the treatment paradigm for this disease [21, 22].

Given the prevalence of pLGGs and the favorable long-term survival outcomes, avoidance of serious long-term treatment-related sequelae is crucial. MN-pCT are not generally associated with pLGG treatment, with only two previous cases reported worldwide to our knowledge [23, 24]. Here we report a novel case of a 17-year old girl who developed MDS-pCT following chemotherapeutic treatment for pLGG.

## Patient presentation

**Fig. 1:**
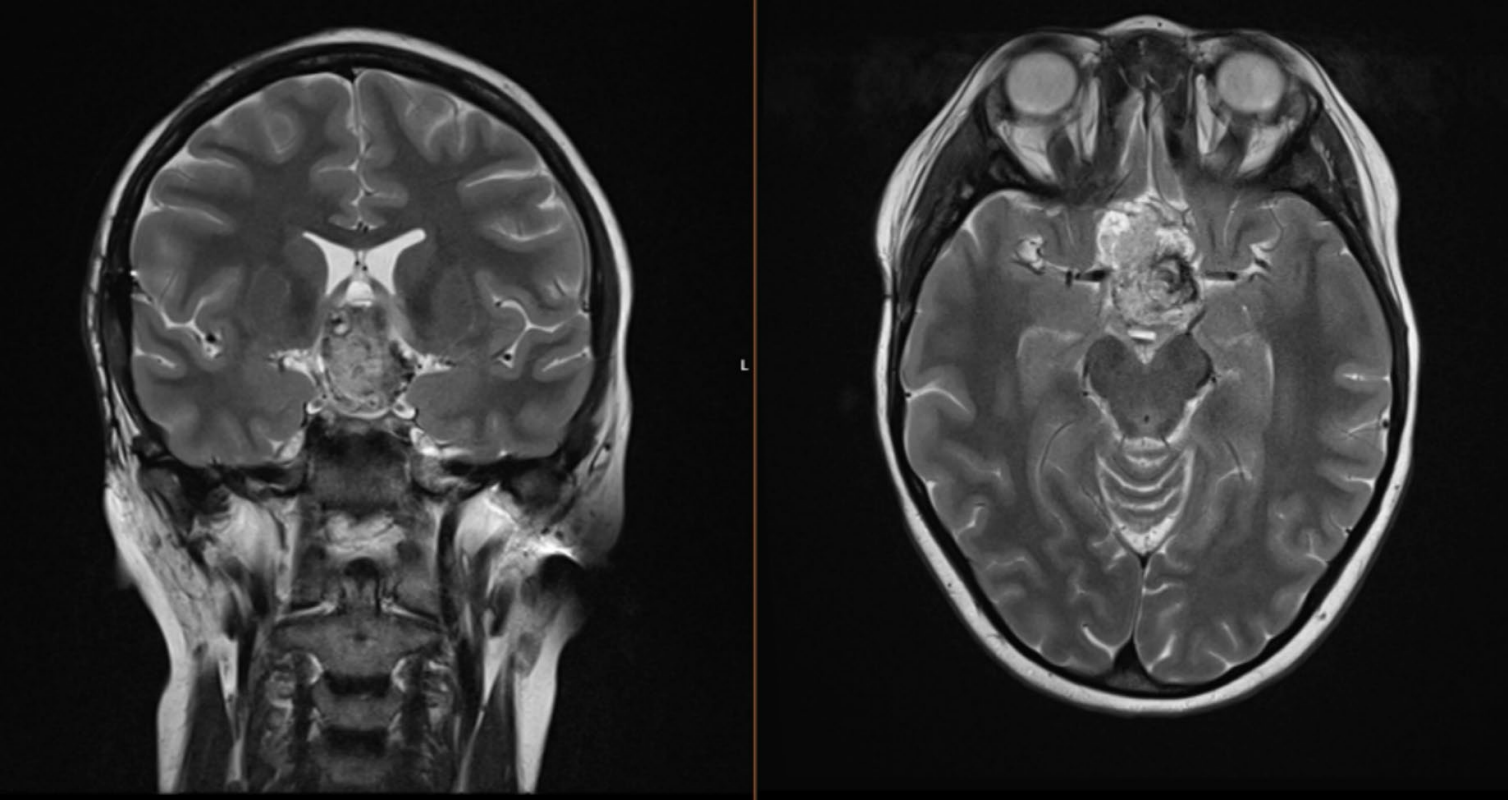
MRI brain (T2 sequence): heterogeneously T2 hyperintense suprasellar lesion measuring 26 × 43 × 29 mm (transverse × AP × craniocaudal)

The patient initially presented at age 4 with diencephalic syndrome. MRI revealed a large suprasellar tumor involving the hypothalamus and optic chiasm and biopsy confirmed a pilomyxoid astrocytoma, WHO Grade 1*.* She was initially treated with chemotherapy as per the COG A9952 Regimen A protocol with weekly vincristine and carboplatin (cumulatively receiving approximately 7 g/m^2 of carboplatin), completing treatment at age 5. She had a year-long period of disease stability before progression at age 7, when she was commenced on second-line chemotherapy treatment per the COG A9952 Regimen B protocol with thioguanine, procarbazine, lomustine and vincristine (cumulatively receiving 1600 mg/m^2 and 880 mg/m^2 of procarbazine and lomustine respectively). After a prolonged period of stability, the patient had slow progression of the suprasellar lesion at age 17, (8 years after the completion of chemotherapy), associated with progressive peripheral visual field impairment (Fig. 1). Given the potential for targeted therapeutic options, the patient underwent a repeat biopsy, which confirmed a characteristic *KIAA1549::BRAF* fusion.

**Fig. 2:**
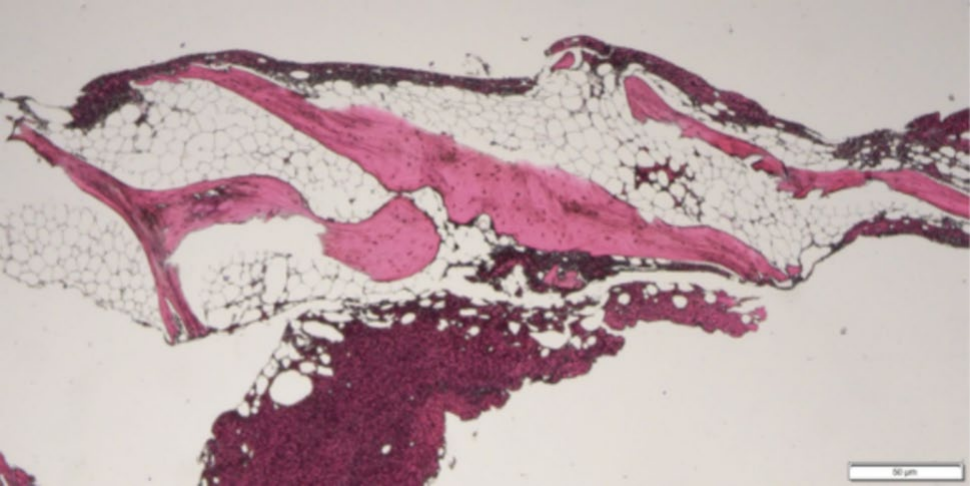
Markedly hypocellular bone marrow for age

**Fig. 3:**
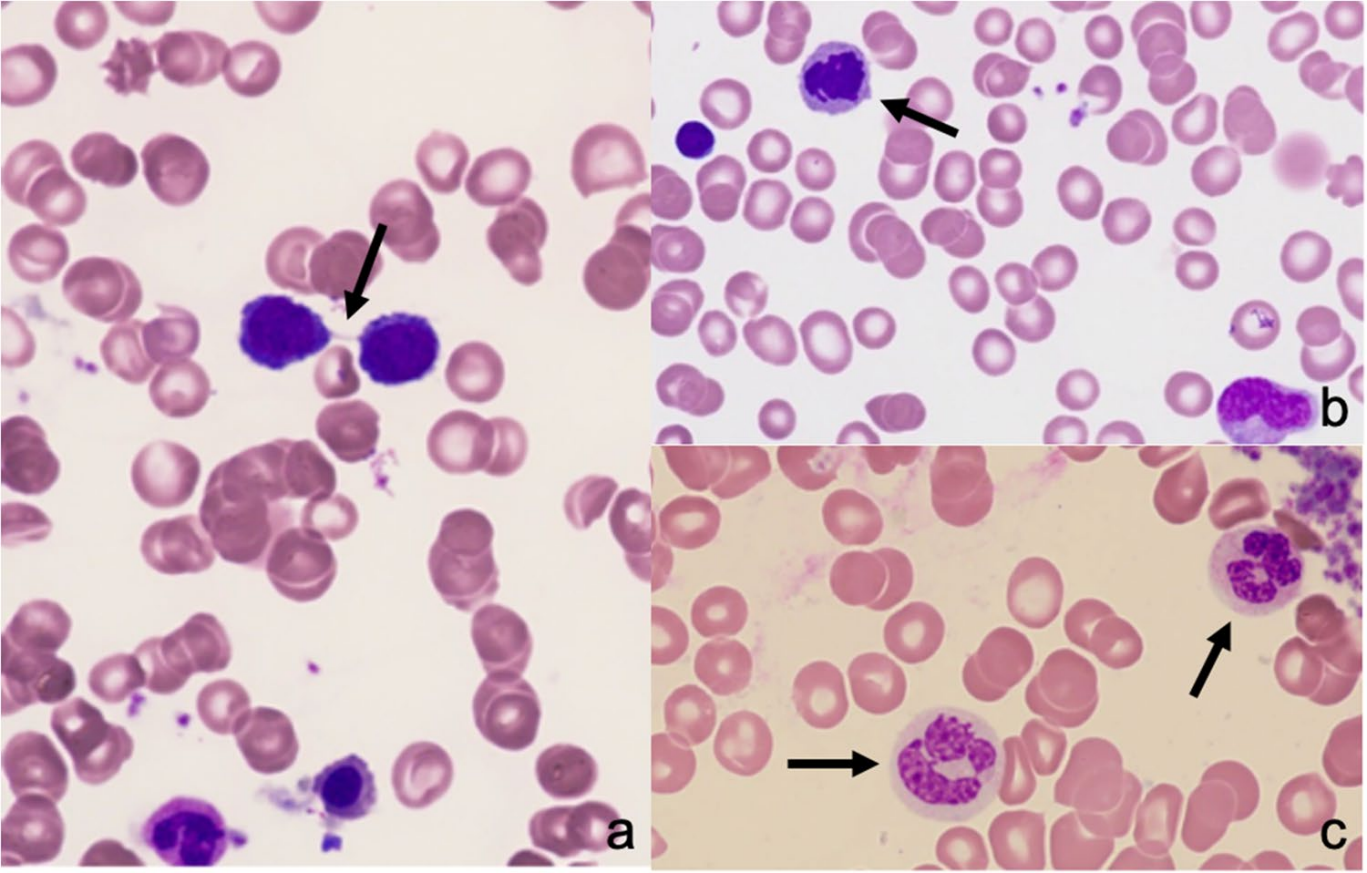
Dysplastic bone marrow changes. **a** Bone marrow dysplasia with internuclear bridging. **b** Karyorrhexis. **c** Hypogranular neutrophil

Prior to commencing treatment with a MAPK pathway inhibitor as third line therapy, the patient was noted to have neutropenia (ANC 0.1–1.1 × 10^9^/L) and macrocytic anemia (Hb 75–95 g/L, MCV of 95–105 fL). This bicytopenia persisted for 6 months, prompting a diagnostic bone marrow biopsy (BMB) which revealed a hypocellular marrow with reduced trilineage hematopoiesis and a clonal cytogenetic abnormality with a gain of chromosome 1 (Table 1). Serial monitoring showed progression of marrow hypocellularity and trilineage dysplasia in > 10% of cells, consistent with myelodysplasia (Figs. 2 and 3). In addition, there was clonal evolution of the cytogenetic abnormalities, with development of three separate abnormal populations (Table 1). Blasts were not increased.

**Table 1:** Serial bone marrow biopsy results

BMB date	Morphologic findings	Cytogenetic findings
Dec 2022^1^	Moderately hypocellular (30–40%) No dysplasia evident	Gain of chromosome 1p in 13% of cells
Feb 2023	Markedly hypocellular (0–10%) Dysplastic changes evidence in < 10% of erythroid and granulocytic precursors	Gain of chromosome 1p in 13% of cells Complex karyotype in 2% cells (trisomy 1, 8 and der(1;18))
Mar 2023	Moderately hypocellular (30–40%) Trilineage dysplastic changes of > 10%, consistent with myelodysplasia	Gain of chromosome 1p in 40% of cells Trisomy 1 and der(1;18) in 4% cells

^1^First BMB performed 9 years after completion of chemotherapy (and 13 years after initial diagnosis)

Of note, germline whole exome DNA next-generation sequencing was done to investigate for any bone marrow failure syndromes or underlying genetic predisposition to increased treatment-related toxicity. No significant germline variants were found.

In the setting of myelodysplasia with clonal evolution and the emergence of new cytogenetic abnormalities, the patient was diagnosed with myelodysplastic syndrome-post cytotoxic treatment (MDS-pCT), 9 years after chemotherapy and 13 years following her original brain tumor diagnosis. She underwent a hematopoietic stem cell transplant, (10/10 matched-unrelated donor, CD34 selected graft, with reduced intensity conditioning regimen of Thiotepa/Fludarabine/ATG). She is now 18 months post-HSCT and is clinically well, with full donor engraftment.

## Discussion

MC-pCT are well known to be associated with alkylating agents (including carboplatin, lomustine, and procarbazine) and topoisomerase II inhibitors, with risk correlating to cumulative dose exposure [2–6, 25]. MN-pCT exhibit a higher proportion of high risk cytogenetic abnormalities than de novo AML, particularly TP53 mutations (found in ~ 30%) and complex karyotypes, as is seen in this patient [3, 4, 26–28].

MN-pCT are exceedingly rare in the pLGG setting. The largest published review of secondary neoplasms (SNs) in pediatric patients with primary CNS tumors retrospectively analyzed 1283 patients; among these, 24 SNs were identified, including 3 patients with t-MN, none of whom had a primary diagnosis of pLGG [29]. There have only been two other reported cases of MN-pCT occurring in LGG patients [23, 24]. The first patient was treated similarly to our case with sequential alkylator-based regimens (vincristine-carboplatin followed by TPCV at recurrence) and developed t-MDS 9 years following diagnosis [23]. Details of the other patient’s treatment are not known [24].

Pediatric MN-pCT carry a poor prognosis with a 5-year OS in patients treated with HSCT of 30% (based on limited pediatric data series) compared with de novo AML which has an expected 5-year overall survival of 75% [2, 3, 6–11, 30]. For patients with a primary diagnosis of pLGG, which carries an otherwise excellent 10-year OS of > 85%, MN-pCT represent an unacceptable secondary treatment effect [12]. Given the evolving understanding of the molecular drivers in pLGG, MAPK-targeted therapies are being increasingly used in this disease, with several agents FDA-approved in the both the upfront and recurrent/progressive settings, and multiple others under investigation in phase II/III clinical trials [21, 22]. Consideration of long-term treatment morbidity is paramount in pLGG, and this case highlights a particularly devastating entity that may prompt consideration of targeted treatment options in an effort to avoid prolonged alkylator exposure and risk of secondary malignancy.

## Data Availability

No datasets were generated or analysed during the current study.
